# Distribution of triatomine species in domestic and peridomestic environments in central coastal Ecuador

**DOI:** 10.1371/journal.pntd.0005970

**Published:** 2017-10-02

**Authors:** Mario J. Grijalva, Anita G. Villacís, Ana L. Moncayo, Sofia Ocaña-Mayorga, Cesar A. Yumiseva, Esteban G. Baus

**Affiliations:** 1 Centro de Investigación para la Salud en América Latina, Escuela de Ciencias Biológicas, Facultad de Ciencias Exactas y Naturales, Pontificia Universidad Católica del Ecuador, Apartado, Quito, Ecuador; 2 Infectious and Tropical Disease Institute, Department of Biomedical Sciences, Heritage College of Osteopathic Medicine, Ohio University, Athens, Ohio, United States of America; National Institute of Allergy and Infectious Diseases, UNITED STATES

## Abstract

**Background:**

Although the central coast of the Ecuador is considered endemic for Chagas disease, few studies have focused on determining the risk of transmission in this region. In this study we describe the triatomine household infestation in Manabí province (Central Coast region), determine the rate of *Trypanosoma cruzi* infection and study the risk factors associated with infestation by *Rhodnius ecuadoriensis*.

**Methodology/Principal findings:**

An entomological survey found three triatomine species (*Rhodnius ecuadoriensis*, *Panstrongylus rufotuberculatus* and *P*. *howardi*) infesting domiciles in 47.4% of the 78 communities visited (total infestation rate of 4.5%). Four percent of domiciles were infested, and nymphs were observed in 77% of those domiciles. The three species were found in altitudes below 500 masl and in all ecological zones except cloud forest. Within the domicile, we found the three species mostly in bedrooms. *Rhodnius ecuadoriensis* and *P*. *rufotuberculatus* were abundant in bird nests, including chicken coops and *P*. *howardi* associated with rats in piles of bricks, in the peridomicile. Triatomine infestation was characterized by high rates of colonization, especially in peridomicile. Flagelates infection was detected in only 12% of the samples by microscopy and *Trypanosoma cruzi* infection in 42% of the examined triatomines by PCR (n = 372). The most important risk factors for house infestation by *R*. *ecuadoriensis* were ecological zone (*w* = 0.99) and presence of chickens (*w* = 0.96). Determinants of secondary importance were reporting no insecticide applications over the last twelve months (*w* = 0.86) and dirt floor (w = 0.70). On the other hand, wood as wall material was a protective factor (*w* = 0.85).

**Conclusion/Significance:**

According the results, approximately 571,000 people would be at high risk for *T*. *cruzi* infection in Manabí province. A multidisciplinary approximation and the adhesion to a periodic integrated vector management (IVM) program are essential to guarantee sustainable preventive and control strategies for Chagas disease in this region.

## Author summary

Chagas disease is an important public health problem in most of Latin America, including Ecuador. It is caused by the parasite *Trypanosoma cruzi*, which in endemic areas is transmitted mainly by the feces of insects called triatomines. Control efforts in the region have reduced the number of new cases of Chagas disease, but there is limited information in certain countries, such as Ecuador. We conducted the first province-wide entomological survey conducted in central coastal Ecuador and report the distribution of triatomines, their habitat and microhabitat preferences, and factors that favor their presence. The results of the study show that 4.5% of households in 78 rural communities harbor triatomine populations. Infestation is higher outside, than inside the houses, but is well established, as shown by the presence of inmature stages and eggs. These results, combined with recent reports of lack of effectiveness of insecticide spraying efforts in this region show the need for innovative approaches for vector control to reduce the high risk of infection to approximately 571,000 people living in rural areas of central coastal Ecuador.

## Introduction

Chagas disease has been recognized as one of the world’s 13 most neglected tropical diseases and continues to be an important social and economic problem in many Latin American countries[1]. About 8–9 million people are estimated to be infected with *Trypanosoma cruzi* in Latin America, including 2–5 millon cases with Chagasic cardiomyopathy[1]. In Ecuador, it is estimated that approximately 200,000 people (1.4%) are affected by this disease and 29.0% are at risk of infection[2].

Sixteen species of insect vectors have been reported in Ecuador and at least 13 are vectors or potential vectors of Chagas disease [3,4]. In Manabí province, five species of triatomines have been reported: *Rhodnius ecuadoriensis*, *Panstrongylus rufotuberculatus*, *P*. *howardi*, *P*. *geniculatus and Triatoma dimidiata* [3]. *Rhodnius ecuadoriensis* is considered one of the most important vector species in Ecuador due to its wide geographic distribution and has been reported along the central coastal region, through southern Ecuador to northern Perú [3,5,6]. This species has the ability to invade domestic, peridomestic, and sylvatic habitats and show high infection rates with the parasite *T*. *cruzi* [7,8]. Sylvatic populations have been found in association with squirrel nests (*Sciurus nebouxii*, previously known as *Sciurus stramineus*)[7,9,10, 31], the endemic palm *Phytelephas aequatorialis* [3] and other plant species [9].

In the coastal region of Ecuador, other triatomine species, including *Panstrongylus howardi* and *P*. *rufotuberculatus*, have shown capacity to infest human dwellings and their surroundings and have been also found naturally infected with *T*. *cruzi* [4,9,11]. However, sylvatic populations are absent for *P*. *rufotuberculatus* [8] or very restricted for *P*. *howardi* [9].

*Panstrongylus howardi* is the second most abundant species in coastal Ecuador and its distribution is restricted to the dry areas of Manabí province in the central coastal region [12]. Taxonomically, this *P*. *howardi* is closely related to *P*. *chinai*, but the specific phylogenetic relationship between them has not been clearly defined [13]. Interestingly, this species has also been misidentified in the field as *T*. *dimidiata* (previously considered the main vector for Chagas disease in Ecuador) due to their similar chromatic pattern [11,14]. In consequence, it is possible that previous data related to the abundance and distribution of this species could be inaccurate. The presence of *P*. *rufotuberculatus* has been restricted to domestic environments from central coast to southern Ecuador (in Santo Domingo de los Tsáchilas, Manabí, El Oro, Los Ríos and Loja provinces) and is widely distributed in Central and South America [3,5,8,9]. Despite the emerging importance of these species in the coastal region of Ecuador, little is known about its distribution and biology. These data are highly required to manage vector control interventions considering the proven limited effectiveness of the delthamethrin spray strategy [11].

*T*. *dimidiata* is present in some areas of the coastal region, mainly in Guayas province[15], and is considered an introduced species [3]. Little is known about *Panstrongylus geniculatus* in Ecuador and this species does not colonize human dwellings which limits its potential as a vector of Chagas disease [16].

Manabí province is located along the central part of Ecuador’s Pacific Coast and has approximately 1,363,285 inhabitants, of which 43.6% live in rural areas [17]. In this province, poverty, defined as “unmet basic needs”, is about 76.8% which exceeds the national average (60.1% for Ecuador). Manabí is the second province with the highest proportion of rural poverty in the country (96.2%) [17]. The main economical activity is agriculture and the principal crops are: cocoa, banana, coffee, corn, rice and cotton. In April 2016, many areas of this province were devastated by the effect of an earthquake.

This study was designed to 1) to describe the geographical distribution of Chagas disease vectors along Manabí province (central coast), 2) to identify the microhabitats occupied by the vector species, 3) to study the risk factors associated with household infestation with the most abundant vectors species, *R*. *ecuadoriensis* and 4) to determine the prevalence of infection with trypanosomes.

## Methods

### Study area and population

This study was conducted in 78 rural communities in 20 of the 22 counties of Manabí province in three visits (2009, 2010 and 2011) during the dry season (July, August and September). This province is located in the central coast of Ecuador and has a climate between subtropical dry and tropical humid. This province experiences two seasons: rainy season (December to May) and dry season (June to November). The average temperature for 2009, 2010 and 2011 was 26.3°C, 25.4°C and 25.5°C, respectively. This province registered an average rainfall of 337.5 mm for 2009, 745.9 for 2010 mm and 355.2 mm for 2011[18,19,20,21]. The main economic activity is agriculture, including sugar cane, cocoa, coffee, bananas, corn, rice, cotton and fruits. In addition, palm plantations (*P*. *aequatorialis*, *Cocos nucifera*, *Elaeis guineensis*) are common in this region. These palms are used to obtain fats and oils, chemicals, paper and in the manufacture of handicrafts and buttons [22]

We visited 3,000 households of which 903 (30.1%) were uninhabited, closed or the owners did not accept to participate in the study. Therefore, the study area included 2,097 households which were located at an altitude ranging from 9 to 1,070 meters above the sea level (masl) and encompassed six vegetation zones: deciduous forest, semi-deciduous forest, green low mountain forest, cloud forest, dry mountain bush forest and tropical savanna.

### Household survey

A cross-sectional survey of 2,097 household in the study area was conducted. This sample size allows detection of infestation indices of 4.5 with a precision of 1%: The location of each site was georeferenced with a GPS receiver (Garmin eTrex Summit), and photos were taken of the four sides of each domicile. To determine the risk factors associated with *R*. *ecuadoriensis* infestation, a questionnaire was presented to the head of each household to obtain information about construction materials of the different parts of the house (floor, wall, roof), presence of toilet, number of inhabitants and bedrooms, number and type of domestic animals they owned and time since self-reported and vector control program insecticide spraying.

### Triatomine collection

Simultaneously with the household survey, domiciles and peridomiciles were searched for triatomine bugs by a modification of the one-person-hour method previously described [5,23] and conducted by two-person skilled teams from the national or provincial vector control programs. As previously described[8], if no bugs were found after the initial 20-min search, the searches were continued for an additional 10 min using of 6% pyrethrins solution (PRENTOX, Excite, Prentiss Inc., New York, USA) as a bug irritant. Collected triatomines were placed individually in labeled plastic containers and transported to either the field laboratory or the insectary at the Center for Research on Health in Latin America at Catholic University in Quito[4,24]. If live triatomines were found in or around the domicile, both environments were sprayed. Spraying was done with 5% deltamethrin WP that was applied at 25 mg/m^2^ by trained personnel from the National Chagas Control program using Hudson X-pert sprayers (H. D. Hudson Manufacturing Co., Houston) [23].

### Natural infection with trypanosomes

Collected triatomines were washed in White’s solution (HgCl 0.8mM, NaCl 111mM, HCl 0.125%, and 25% v/v of ethanol 95%) before being dissected under a stereo microscope. Feces and intestinal content were mixed with 200μl of sterile PBS. One 150μl aliquot was used for microscopic examination to detect flagellates and another aliquot was stored at -20°C for DNA extraction. DNA was obtained with a DNeasy kit (Qiagen, Valencia, CA) following manufacturer´s protocol. The presence of trypanosomatid DNA was determined by PCR amplification using the S35/S36 primer set [25], which amplifies the conserved domain of the minicircle of the kinetoplast DNA (kDNA) and allows discrimination between *T*. *cruzi* and *T*. *rangeli* due to differential band size. The infection index (100 x number of infected individuals/total number of analyzed individuals) was calculated for each species and habitat.

### Data analysis

We describe the distribution of each triatomine species by ecological zone and altitude. A household was considered infested when at least one live triatomine nymph or adult was found and the following entomological indices were calculated: Infestation rate (100 x number of houses infested /number of houses searched), density (number of triatomines captured/number of houses searched), crowding (number of triatomines captured/number of houses infested), and colonization index (100 x number of houses with nymphs/number of houses infested) [26].

A multimodel inference approach based on Akaike’s Information Criterion was used to identify the strongest determinants for house infestation by *R*. *ecuadoriensis* according to Burnham and Anderson [27]. First, we fit two subset of models: 1) only household level covariates (floor, wall and roof materials, presence of toilet/latrine, crowding, number of bedrooms, time since self-reported and vector control program insecticide spraying); 2) only domestic animals covariates (number of chickens, dog, cats and guinea pigs). Second, we selected variables of each subset of models according their relative importance (*w*>0.35) for predicting house infestation in order to fit the final model. Ecological zone was included in the final model. The final model considering every possible combination of selected variables was run: 6 variables gave 64 models for house infestation by *R*. *ecuadoriensis*.

The Akaike’s weight (*w*_*i*_) of each model was calculated as the quotient of the log-likehood of the particular model divided by the total sum of the log-likehood of all considered models. The relative importance of a particular variable (*w*) was then calculated as the sum of Akaike weights of all models that contained that particular variable. Variables with *w*>0.9 were considered of high importance in defining house infestation, variables with 0.7<*w*<0.9 were considered of secondary importance and variables with *w*<0.7 had limited contributions.

Finally, in order to obtain a model including the most complete information and the best predictive ability, we performed model averaging to estimate weighted mean effect-sizes estimates (Odds Ratio) resulted from averaging the parameter value in each model where the variable was present weighted by the Akaike weight of the respective model.

Descriptive analysis was assessed in the software STATA v.11.0. Multimodel inference and model averaging were performed in the software R (v. 2.7.0). Data is publically available in S2 Table.

### Ethics statement

The protocol was approved by the institutional review boards of Ohio University and Catholic University of Ecuador. Written informed consent was obtained from the head of the household. Triatomines were collected under Ecuadorian collection permits (2009- N° 016–07 IC-FAU-DNBAPVS/MS; 2010- N°006RM-DPM-MA; 2011- N° 008RM-DPM-MA)

## Results

### Distribution of triatomines by species, ecological region and altitude

**Fig 1:**
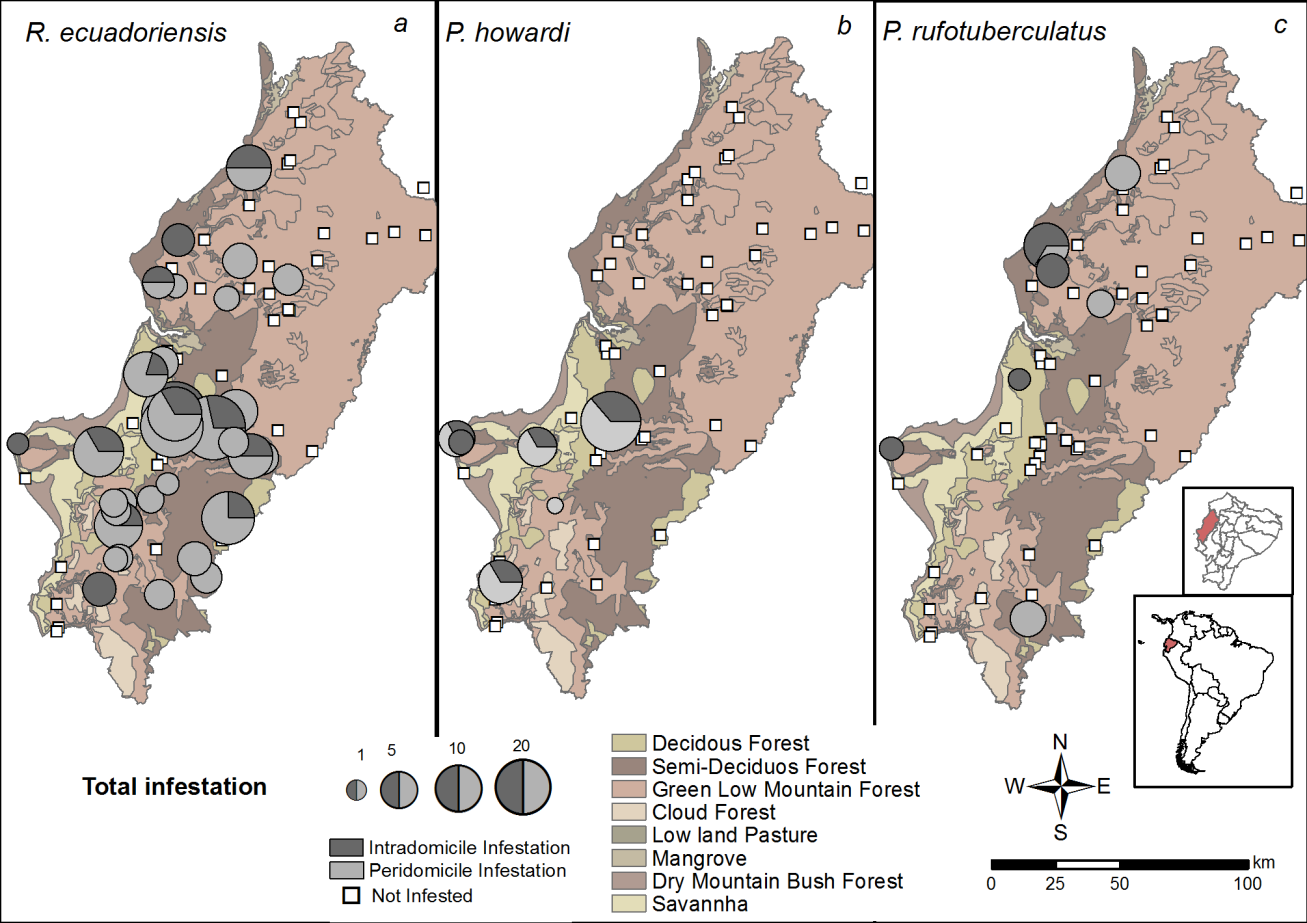
Triatomine infestation in Manabí province, central coast of Ecuador encompassing five ecological regions. Location of each community surveyed is marked. The size of the symbol corresponds to the % of houses infested with triatomines in each community with each of the three triatomine species found (A = *R*. *ecuadoriensis*, B = *P*. *howardi* and C = *P*. *rufotuberculatus*). Dark grey and light grey represent intradomicicle and peridomicile infestation, respectively and white squares represent non-infested communities. Inserts indicate the location of Manabí province within Ecuador and of Ecuador in South America.

Of the 78 rural communities visited, 37 (47.4%) were infested (S1 Table). The most common species was *R*. *ecuadoriensis* which was found in 32 of the 37 infested communities (86.5%). This species was found in communities located in altitudes between 45–471 masl and encompassing four ecological zones (Fig 1): deciduous forest (75% of 8 searched communities), dry mountain bush forest (50% of two searched communities), semi-deciduous forest (44.4% of 18 searched communities) and green low mountain forest (37.8% of 45 searched communities). Less abundant species, *P*. *rufotuberculatus* were found in 18.9% of infested communities at altitudes ranging 45–471 masl and in four ecological zones: dry mountain bush forest (50%), deciduous forest (12.5%), green low mountain forest (8.9%), semi-deciduous forest (5.6%). Finally, *P*. *howardi* was found in 16.2% of infested communities and its distribution reached altitudes between 78–355 masl and five ecological zones: dry mountain bush forest (50%), deciduous forest (25%), savanna (25%), semi-decidous forest (5.6%) and green low mountain forest (2.2%). In addition, we found communities with simultaneous infestation by two or three triatomine species: presence of *R*. *ecuadoriensis* and *P*. *howardi*, and *R*. *ecuadoriensis* and *P*. *rufotuberculatus* was reported in 10.8% of infested communities while 2.7% reported infestation by the three species (Fig 1).

### Infestation indices

There were 1380 live insects collected and 114 eggs. Only live triatomines were included in the analysis. Among the 2097 searched households, 95 (4.5%) were infested by at least one triatomine species. The density was 0.7 bugs per examined domicile, the crowding was 14.5 bugs per infested domicile and the colonization index was 76.8% (Table 1).

**Table 1:** Entomological indices of triatomine infestation by species and habitats in rural communities of Manabí province, 2009–2011.

Species	Number of houses searched	Entomological indices						
		Infested domicilies	Live Triatomines collected	Houses with nymphs	Infestation (%)	Density	Crowding	Colonization (%)
**Intradomicile**								
*R*. *ecuadoriensis*	2097	14	121	6	0.7	0.1	8.6	42.9
*P*. *howardi*	2097	8	8	1	0.4	0.0	1.0	12.5
*P*. *rufotuberculatus*	2097	5	29	3	0.2	0.0	5.8	60.0
**Total**	2097	26	158	10	1.2	0.1	6.1	38.5
**Peridomicile**								
*R*. *ecuadoriensis*	2097	59	1086	57	2.8	0.5	18.4	96.6
*P*. *howardi*	2097	14	80	9	0.7	0.0	5.7	64.3
*P*. *rufotuberculatus*	2097	4	56	3	0.2	0.0	14.0	75.0
**Total**	2097	73	1222	64	3.5	0.6	16.7	87.7
**All habitats**								
*R*. *ecuadoriensis*	2097	73	1207	63	3.5	0.6	16.5	86.3
*P*. *howardi*	2097	20	88	10	1.0	0.0	4.4	50.0
*P*. *rufotuberculatus*	2097	8	85	5	0.4	0.0	10.6	62.5
**Total**	**2097**	**95**	**1380**	**73**	**4.5**	**0.7**	**14.5**	**76.8**

Infestation indices were low (under 10%) in most localities (78.4%), but were significantly greater in El Bejuco (29.7%), Portoviejo County; Danzarin (18.8%), Rocafuerte County and Tablada de Algodon (18.4%), Junín County (S1 Table). Analysis of density and crowding indicated that Dislabon, Chone County, had the highest values (6.5 bugs per searched domicile and 123 bugs per infested domicile, respectively). A 100% colonization index was observed in 23 of 37 infested communities (62.2%).

### Species-specific entomological indices and habitat

Of the 1,380 insects collected, 1,222 (88.5%) were found in the peridomestic habitat. Overall, all entomological indices were higher in the peridomicile when compared with the domicile (Table 1). *Rhodnius ecuadoriensis* showed an infestation index in the peridomicile 2.1 times higher than in the domicile. The other species showed little or no difference on the infestation index between domicile and peridomicile habitats. The most remarkable differences between domicile and peridomicile habitats were found for crowding and colonization indices for all species (Table 1).

### Species-specific microhabitat preferences

*Rhodnius ecuadoriensis* was more often collected in chicken nests (44.3%) and rat nests (36.6%) in the peridomicile while in the intradomicile was mainly found in or near the beds (71.9%; 95% CI 63.0–79.7) and indoor chicken nests (23.1%; 95% CI 16.0–31.7). On the other hand, *P*. *howardi* showed preference for bricks in the peridomicile (81.3%; 95% CI 71.0–89.1) and in the intradomicile was mainly collected in or near the bed (42.8%; 95% CI 9.8–81.6) and in the bedroom wall (28.6%; 95% CI 3.7–71.0). Finally, *P*. *rufotuberculatus* was found mostly in bird nests other than chicken nests (44.6%; 95% CI 31.3–58.5) and in chicken nest (32.1%; 95% CI 20.3–46.0) in the peridomicile. In the domicile, this species showed preference for microhabitats located in or near the beds (96.5%; 95% CI 82.2–99.9).

### Species specific population structure

**Fig 2:**
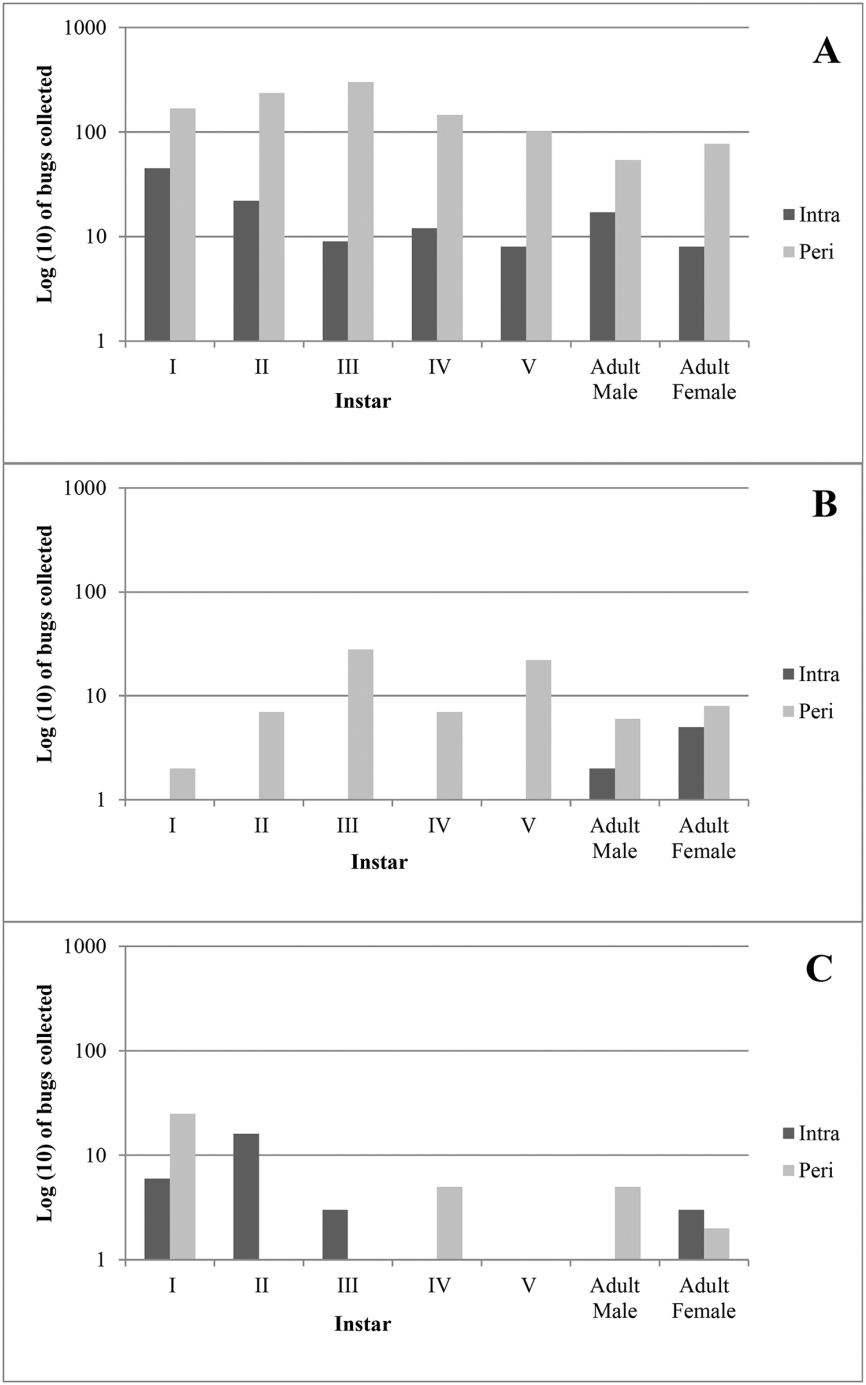
Population structure of triatomines collected in rural communities of Manabí province, Ecuador. Number of live Nymphal I-V and adult female and male bugs collected in domestic (Intra) and peridomestic (Peri) habitats. A) *R*. *ecuadoriensis*, B) *P*. *howardi* and C) *P*. *rufotuberculatus*.

More abundance of *R*. *ecuadoriensis* and *P*. *howardi* nymphs was found in peridomicile (*R*. *ecuadoriensis*: 90%, 95% CI 88.1–91.6; *P*. *howardi*: 90.9, 95% CI 82.9–96.0) than in domicile (*R*. *ecuadoriensis*: 10%; 95% CI 8.4–11.9; *P*. *howardi*: 9.1%, 95% CI 4.0–17.1) (Fig 2). For *R*. *ecuadoriensis*, more instar II (21.8, 95% CI 19.4–24.4) and III (27.8, 95% CI 25.2–30.6) were collected in the peridomicile while in the domicile more nymphs I (37.2, 95% CI 28.6–46.4) and II (18.2, 95% CI 11.8–26.2). For *P*. *howardi*, most instars in the peridomicile were III (35.0, 95% CI 24.7–46.5) and V (27.5, 95% CI 18.1–38.6) while in the domicile only one nymph IV was collected. For both species more female than male adults were found in the peridomicile. On the contrary, *P*. *rufotuberculatus* nymphs II and III were not collected in the peridomicile but more abundance of nymphs I (65.8, 95% CI 48.6–80.4) was observed in this habitat (Fig 2). In the domicile, most nymphs were instar II (55.2, 95% CI 35.7–73.5). For this species, the number of adult males was higher than females in the peridomicile and the opposite in the domicile.

### Characteristics of domiciles

We searched 2,097 houses for the presence of triatomines. The Table 2 shows the characteristics of domiciles. The vast majority of houses had walls constructed with canes (50.4%), cement/asbestos/zinc for the roof (91.6%) and wood for the floor (74.4%). The 81.2% and 71.8% reported to have toilet and less than 2 bedrooms per house, respectively. A low percentage of families reported that their houses have been sprayed in the last 12 months by themselves (31.2%) or by the vector control program (5.6%). The majority of domiciles had chickens (91.3%) and dogs (74.5%). Cats (34.7%) and guinea pigs (*Cavia porcellus*) (3.3%) were less common (Table 3).

**Table 2:** Characteristics of domiciles from 78 rural communities in Manabí province.

House characteristics	N 2,097	%
**Roof material**^**(21)**^		
Cement/asbestos/zinc	1,901	91.6
Tile	13	0.6
Palm, other	162	7.8
**Wall material**^**(23)**^		
Cement/brick2	602	29.0
Adobe/bahareque	5	0.2
Wood	421	20.3
Cane, other	1,046	50.4
**Floor**^**(23)**^		
Cement/title/wooden parquet	386	18.6
Wood boards	1,542	74.4
Cane, other	87	4.2
Dirt	59	2.8
**Size and inhabitants**		
Bedrooms ≤2^(22)^	1,490	71.8
Inhabitant crowding^(26)^	456	22.0
**Toilet**^**(26)**^		
Yes	1,682	81.2
No	389	18.8
**Insecticide Sprayed**		
Self reported <12 months^(22)^	647	31.2
Vector Control Program <12 months^(28)^	116	5.6

Numbers of missing values are given in parentheses

Inhabitant crowding = more than 3 people per bedroom

**Table 3:** Livestock found in 78 rural communities of Manabí province.

Livestock	Number of houses	%	Animals per domicile median (IQR)
Chickens, other birds^(19)^	1,897	91.3	20 (10–30)
Dogs^(20)^	1,547	74.5	2 (1–3)
Cats^(20)^	723	34.7	1 (1–2)
Guinea pigs (outdoor)^(20)^	68	3.3	3 (2–4)

IQR = Interquartile Range

### Determinants of *R*. *ecuadoriensis* infestation

The household-covariate model set included 128 models and the best supported model indicated that construction materials of wall (*w* = 0.94), self-reported insecticide spraying (*w* = 0.86), dirt floor (w = 0.58) and vector control program insecticide spraying (*w* = 0.36) were relatively more important than construction material of the roof (*w* = 0.29), number of bedrooms (*w* = 0.28) and crowding (*w* = 0.28).

In the next step, we fit all 16 possible model specifications including the four domestic animals covariates. According to their relative importance, only chickens (*w* = 0.92) were retained for further analysis, whereas dogs (*w* = 0.16), cats (*w* = 0.14) and guinea pigs (*w* = 0.13) were eliminated.

We then proceed to model averaging to identify the strongest determinants for house infestation by *R*. *ecuadoriensis*. Table 4 presents weighted averaged effect size over all models (6 variables, 64 models) in the set for each of these covariates. The relative importance of each variable in the averaged model indicated that two variables were the most relevant factors positively associated with *R*. *ecuadoriensis* infestation (*w*>0.9). These variables include ecological zone (*w* = 0.99) and presence of chickens (*w* = 0.96). In addition, households located in the semi-deciduous and deciduous forest showed 1.6 and 3.3 higher risk of infestation, respectively, when compared to households located in dry mountain forest (reference category). Secondary predictors that increase the risk of infestations were self-reported insecticide spraying for more than 12 months or never (*w* = 0.86) and dirt floor (*w* = 0.70). On the other hand, wood wall decreased the risk of infestation (*w* = 0.85).

**Table 4:** Important determinants for house infestation by *R*. *ecuadoriensis* in Manabí province, 2009–2011.

Factor	Total	Infested DUs %	OR*	SE	RI (*w*)
**Ecological zone**					0.99
Dry mountain bush forest	57	1.75	1.00		
Green low mountain forest	1,231	2.68	1.17	1.06	
Semi-decidous forest	428	3.97	1.59	1.07	
Deciduous forest	263	8.37	3.29	1.05	
Cloud forest**	12	0.00	——		
Tropical Savanna**	87	0.00	——		
**Chickens, other birds**					0.96
none	181	0.55	1.00		
<20	901	3.22	6.40	1.03	
≥ 20	996	4.32	9.45	1.02	
**Self Reported Insecticide Spray**					0.86
<12 months	647	2.16	1.00		
≥12 months/none	1,431	4.12	1.98	0.31	
**Wall material**					0.85
Cement/brick	602	3.82	1.00		
Wood	421	1.19	0.34	0.52	
Cane, other	1,055	4.27	1.07	0.27	
**Dirt floor**					0.70
No	2,019	3.37	1.00		
Yes	59	8.47	2.98	0.51	
**Vector Control Program Insecticide Spray**					0.28
<12 months	116	2.59	1.00		
≥12 months/none	1,962	3.57	1.23	0.61	

*Model_averaged effect-sizes (OR) from the final 64 -model set.

OR: Odds Ratio; SE: standard error

RI: Relative Importance of variables was assessed by multi-model inference based on Akaike´s information criterion.

**Cannot be reliably estimated because of small numbers

### Natural trypanosome infection of triatomines

A total of 372 intestinal content samples were analyzed from domicile (n = 60) and peridomicile (n = 312). The samples cover 28 communities in 18 counties. The microscopic analysis detected only 12% of samples with flagellates. However, molecular analysis determined that the infection with *T*. *rangeli* did not exceed the 9%. *Rhodnius ecuadoriensis* was the species with highest infection rate (10%), while infection with *T*. *cruzi* was detected in 42% of the samples (Table 5). Only one sample presented mix infection with both species of *Trypanosoma* (0.4%).

**Table 5:** Infection rates with *T*. *cruzi* and *T*. *rangeli* in triatomines collected in domicile and peridomicile habitats in Manabí province, 2009–2011.

Species	Domicile		Peridomicile		Total		
	*T*. *cruzi*	*T*. *rangeli*	*T*. *cruzi*	*T*. *rangeli*	*T*. *cruzi*	*T*. *rangeli*	N
	n (%)	n (%)	n (%)	n (%)	n (%)	n (%)	
*R*. *ecuadoriensis*	23 (62)	2 (5)	104 (39)	29 (11)	127 (42)	31 (10)	304
*P*. *howardi*	3 (50)	-	20 (71)	1 (4)	23 (68)	1 (3)	34
*P*. *rufotuberculatus*	2 (12)	-	6 (35)	2 (12)	8 (24)	2 (6)	34
**Total**	**28 (47)**	**2 (3)**	**130 (42)**	**32 (10)**	**158 (42)**	**34(9)**	**372**

n = number of specimens

All three species of triatomines presented high infection rate with *T*. *cruzi* (>20%). *P*. *howardi* despite not being the most abundant species, reported the highest infection rate (68%). While the abundant *R*. *ecuadoriensis* presented 42% of *T*. *cruzi* infection (Table 5). *Panstrongylus rufotuberculatus* presented the lower infestation rate and abundance in houses, however a high infection rate with *T*. *cruzi* was detected (23%, n = 34) (Table 5).

In general, a high infection rate with *T*. *cruzi* (>40%) was reported in the domicile and peridomicile (Table 5). More *R*. *ecuadoriensis* were found to be infected with *T*. *cruzi* in the domicile than in the peridomicile (chi-square test: *p* = 0.007). Although the two species of *Panstrongylus* presented higher infection rates in the peridomicile that in the domicile, the differences were not statistically significant (Fisher´s exact test: *P*. *howardi*; *p* = 0.363 and *P*. *rufotuberculatus; p* = 0.225). On the other hand, the presence of *T*. *rangeli* in the peridomicile is more frequent (10%) than in the domicile (3%) (Table 5).

## Discussion

The most abundant species in the domicile and peridomicile of househols in Manabí province was *R*. *ecuadoriensis*. Less abundant species were *P*. *rufotuberculatus* and *P*. *howardi*. The three species were found in altitudes below 500 masl and in all ecological zones except cloud forest. Infestation index were low (under 10%) in most localities (78.4%) but was found higher than 20% and 10% in some communities of Portoviejo, Junin and Rocafuerte Counties. High rates of colonization were observed indicating well adapted bug populations.

These findings confirm the distribution and abundance of *R*. *ecuadoriensis* in the Central Coast of Ecuador; however, no populations of *T*. *dimidiata* and *P*. *geniculatus* were found despite of the extensive collection effort. Previous reports of triatomines distribution in Ecuador indicated that the main vector species of Chagas disease in the Pacific slope of the Ecuadorian Andes were *Triatoma dimidiata* and *Rhodnius ecuadoriensis* [3,14]. Apparently, the populations of *T*. *dimidiata* are restricted to urban areas of the Coastal region, especially in Guayaquil city, Guayas province and Portoviejo city, Manabí province[15].

*Rhodnius ecuadoriensis* has been found infesting, primarily, peridomestic and sylvatic habitats in the Central Coast region of Ecuador [11]. In the peridomestic habitat, this species has been reported in chicken nests, guinea pig pens, wood piles and rat nests [11]. Of all domestic animals included in our study, only the presence of chickens was retained as an important factor for *R*. *ecuadoriensis* infestation, although chickens (91.3%) and dogs (745%) were very abundant. Given that dogs and cats have been reported as important reservoirs hosts and sentinels for *T*. *cruzi* along the continent [28,29,30], more studies are required to understand their involvement in *T*. *cruzi* transmission in Ecuador. In the sylvatic habitat, *R*. *ecuadoriensis* has been associated with the endemic palm *Phytelephas aequatorialis* [3] and the rodent *Sciurus nebouxii* (previously known as *Sciurus stramineus*) [9,31], both present along the province [3,5]. The palm *P*. *aequatorialis* is found in the semi-deciduos forest and households located in the semi-deciduous and deciduous forest showed higher risk of *R*. *ecuadoriensis* infestation in our study. This palm is cultivated along the province for its nuts used in the manufacture of handicrafts and buttons [22] and its abundance in peridomiciles may have an impact in the limited success of insecticide-based control strategy because palm tree triatomine populations could readily invade treated houses when residual insecticide activity declines [11]. Moreover, the high colonization rates in domicile and peridomicile indicates probably a long term process of the domestication of *R*. *ecuadoriensis*.

According to Costales et al. (2015) [32], the only Discriminant Typing Unit (DTU) of *T*. *cruzi* reported in the Central Ecuadorian Coast is TcI. There is high connectivity between transmission cycles (sylvatic vs. peridomestic and domestic habitats) [32] with a parasite dispersal between sylvatic and peridomestic environments higher than in Loja province were *R*. *ecuadoriensis* also predominates [33]. The presence of *T*. *rangeli* in this area has been previously reported by Grijalva et al. 2011 [11]. In the current study, a low *T*. *rangeli* infection rate was observed in the three species of triatomines. However, *R. ecuadoriensis* is the species with the higher *T*. *rangeli* infection rate (10%). Association of this parasite with *Rhodnius* spp. is well known [34,35,36,37]] while the presence of *T*. *rangeli* in the genus *Panstrongylus* might be the result of temporary infections and do not imply transmission of *T*. *rangeli* by this species [37]. It is important to note that only intestinal content was analyzed and not salivary glands, so the infection rate of *T*. *rangeli* is probably under-estimated.

*Panstrongylus howardi* has a very limited distribution (restricted to Manabí province) [12,14]; however, it has probably an important role in Chagas disease transmission. This species has been reported as one of the two main species infesting peridomestic, domestic, and sylvatic habitats in Manabí province [3,9,11,12]; however little is known about its ecology and life cycle. Although previous studies limited the distribution of *P*. *howardi* to wet forest [13], our study demonstrated the presence of this species in five ecological zones including dry mountain bush forest, deciduous forest, savanna, green low mountain forest and semi-deciduous forest. In the peridomicile, this vector species has been previously found near nests of rats, mice and bullfrogs (*Rana catesbiana*) [9,11] and in piles of bricks, wood and garbage [11]. Accordingly, *P*. *howardi* was more abundant in piles of bricks (potential refuge for rats and mice) than in other habitat. The scarce number of nymphs in the domicile indicates a low colonization success. However, the occurrence of this species in the peridomicile and its high infection rate with *T*. *cruzi* (71%) highlights the need of surveillance of this secondary vector that could increase when the primary vectors (i.e. *Rhodnius ecuadoriensis*) is displaced.

Earlier reports of *P*. *rufotuberculatus* suggested that this species has been primarily found in sylvatic habitats [5,13]; however, high colonization indices in domicile (60%) and peridomicile (75%) were observed as well as the presence of nymphal stages (especially in the domicile) that indicates colonization. The present data confirm the wide distribution of this species encompassing all ecological zones except cloud forest in Manabí province [13]. Besides its low infestation rate (0.4%) and density, the presence of *T*. *cruzi* was confirmed (24%) in domestic and peridomestic environments indicating its importance in maintaining the parasite circulating in the area.

The walls and floor of houses in Manabí province are principally constructed with bamboo cane, *Guadua angustifolia*, or wood and these materials do not allow the triatomines to take refuge because they form very open-air structures. For this reason wood as wall material was found as protective factor for *R*. *ecuadoriensis* house infestation. A previous study reports that roof is commonly made of the cade palm leaves, *Phytelephas aequatorialis*, and in less proportion made of zinc [38]. This is not the case for the present survey where only 7.8% of the houses have palm roof and this material was not retained as a risk factor of infestation although it favors the establishment of the triatomine species. In this context, the structure of the house do not influence the infestation that is more likely due to sporadic visits of *R*. *ecuadoriensis* and *P*. *howardi* from their natural habitats, but multiple microhabitats in peridomiciles allow the colonization of triatomines. A secondary predictor that increases the risk for *R*. *ecuadoriensis* infestation was self-reported insecticide spraying for more than 12 months or never. As mention in a previous study in this region, the intervention with insecticide was not an effective approach for controlling peridomestic triatomine populations in the coastal province of Manabí due to operational issues, among other factors, but could have some effect in domiciles. In our study, infestation index for *R*. *ecuadoriensis* was higher in peridomicile than domicile as a likely effect of insecticide applied for inhabitants.

### Overall implications for public health and control effort

Chagas disease is known as a disease of poverty in rural Latin America. A previous study reported a seroprevalence of 1.99% in the coastal region [39]; however, no systematic seroprevalence studies have been conducted in Manabí Province. According to national census conducted in Ecuador in 2010 [17], Manabí province has 594,855 inhabitants living in rural areas below 500 masl where they are exposed to a high risk of *T*. *cruzi* transmission because of the presence of triatomines in houses and peridomiciles.

The vector control program started in 2004, but important variations in the geographic coverage of the surveillance and control activities were observed year to year due to limited human and financial resources [40]. For example, during the study time, the intervention with insecticide was only focused in two of twenty two counties (Portoviejo and Santa Ana counties) of Manabí province. In recent years, most efforts of surveillance and vector control activities have ceased as a result of elimination of the National Chagas Disease Control Program and *Servicio Nacional de Control y Vigilancia de Enfermedades Transmitidas por Vectores Artrópodos (SNEM)* in 2015. To our knowledge their actions have not yet been replaced [40].

Another serious problem is the effectiveness of insecticide treatment of houses. A previous study in Manabí province showed similar entomological indices before and after selective deltametrin treatment of infested houses indicating little or no effect of the intervention in controlling peridomestic triatomines [11]; the predominance of young nymphs and adults in the reinfestant population strongly suggested their sylvatic origin. The present stategy (insecticide spraying) may be effective against domiciliated and introduced species such as *Triatoma dimidiata*, however, alternative control strategies are needed against native triatomine species such as *Rhodnius ecuadoriensis* and *Panstrongylus howardi*, which maintain sylvatic populations in Manabí province. The previously reported failure of insecticide treatments could be due, among other reasons mentioned above, to ineffective insecticide application, shortened insecticide residual effect due to weather conditions, or high reinfestation pressure from widespread sylvatic triatomine populations [9,11,41]. These results showed that it is necessary to develop alternative methods to improve insecticide application effectiveness in peridomicile and prevent triatomine infestation in regions where well established sylvatic triatomine populations are present. In addition, it is necessary to evaluate the potential for other insecticide-based strategies, such as impregnated bednets, as an alternative for *R*. *ecuadoriensis* vector control. In Perú, a field trial strongly suggests that insecticide-treated nets prevent triatomine bites and can thereby protect against infection with *T*. *cruzi*[42]

Knowledge about Chagas disease and its vectors was very low among the population at the beginning of the project [11]. Therefore, community education should be an integral part of future control efforts. Although tritomine searches in households are highly disruptive to the inhabitants, the educational talks at each household were well received.

A multidisciplinary approximation and the adhesion to Integrated Vector Management (IVM) is essential to address this disease, which include the articulation of health, education, infrastructure, income generation and social organization in order to guarantee sustainable vector and disease preventive and control measures in agreement with local livelihoods. The improvement of the socioeconomic conditions of rural communities could enable access to better living conditions. This, in turn, could reduce risk factors for triatomine infestations. Moreover, an innovative approach is necessary in the environmental management of the disease, which includes improvement of the house structure and the peridomicile. Finally, seroprevalence and congenital transmission studies need to be conducted to better understand the epidemiology of the disease in this region of Ecuador.

## Supporting information

S1 Table Entomological indices and altitude range of triatomine infestation in rural communities of Manabí province, 2009–2011 (37 infested communities). (DOCX)

S2 Table Publically available database. (ZIP)
