# Supplementary material for: Distribution of triatomine species in domestic and peridomestic environments in central coastal Ecuador
Source: PLoS Negl Trop Dis. 2017 Oct 2;11(10):e0005970. doi: 10.1371/journal.pntd.0005970 (PMC5638615; doi:10.1371/journal.pntd.0005970)
Supplement: S1 Table — (DOCX) [file pntd.0005970.s001.docx]

| **S1 Table. Entomological indexes and altitude range of triatomine infestation in rural communities of Manabí Province, 2009-2011 (37 infested communities)** | | | | | | | | | | |
| --- | --- | --- | --- | --- | --- | --- | --- | --- | --- | --- |
|  |  |  |  |  |  |  | |  | | |
|  |  |  |  | **Infestation Index (% colonization)** | | | | | | |
| **Community** | **Number of houses searched** | **Altitude min and max (masl)** | **Live Triatomines collected (No.)** | ***R. ecuadoriensis*** | | | ***P. howardi*** | | ***P. rufotuberculatus*** | **Total** |
| **24 de Mayo County** |  |  |  |  | | |  | |  |  |
| Callejón Santa Rita | 31 | 98 | 37 | 3.2 (100) | | |  | |  | 3.2 (100) |
| **Bolivar County** |  |  |  |  | | |  | |  |  |
| Murucumba | 12 | 418 | 4 | 8.3 (100) | | |  | |  | 8.3 (100) |
| **Chone County** |  |  |  |  | | |  | |  |  |
| Daca | 36 | 205-240 | 22 | 2.8 (50) | | |  | | 2.8 (100) | 5.6 (100) |
| Dislabon | 19 | 133 | 123 | 5.3 (100) | | |  | |  | 5.3 (100) |
| **Flavio Alfaro County** |  |  |  |  | | |  | |  |  |
| Las Canas | 24 | 291 | 2 | 4.2 (0) | | |  | |  | 4.2 (100) |
| **Jama County** |  |  |  |  | | |  | |  |  |
| Estero Seco | 26 | 123-133 | 27 |  | | |  | | 7.7 (50) | 7.7 (50) |
| Punta Larga | 21 | 96 | 1 | 4.8 (0) | | |  | |  | 4.8 (0) |
| **Jipijapa County** |  |  |  |  | | |  | |  |  |
| Canitas | 28 | 203 | 1 | 3.6 (100) | | |  | |  | 3.6 (100) |
| Chande | 58 | 333-375 | 55 | 10.3 (83.3) | | |  | |  | 10.3 (83) |
| La América | 62 | 376-405 | 7 | 3.2 (100) | | |  | |  | 3.2 (100) |
| Mariscal Sucre | 38 | 364 | 12 | 2.6 (100) | | |  | |  | 2.6 (100) |
| Pepa de Uso | 20 | 401 | 1 | 5.0 (0) | | |  | |  | 5.0 (0) |
| San Francisco | 79 | 389-327 | 181 | 3.8 (100) | | | 1.3 (100) | |  | 3.8 (100) |
| Sandial | 29 | 299 | 15 | 3.4 (100) | | |  | |  | 3.4 (100) |
| **Junin County** |  |  |  |  | | |  | |  |  |
| Moralito | 19 | 57 | 2 | 5.3 (100) | | |  | |  | 5.3 (100) |
| Tablada De Algodón | 38 | 424-441 | 153 | 18.4 (100) | | |  | |  | 18.4 (100) |
| **Manta County** |  |  |  |  | | |  | |  |  |
| Liguiqui | 48 | 81-97 | 5 | 2.1 (0) | | | 6.3 (0) | | 2.1 (0) | 8.3 (0) |
| Pacoche | 34 | 207 | 1 |  | | | 2.9 (0) | |  | 2.9 (0) |
| **Montecristi County** |  |  |  |  | | |  | |  |  |
| Santa Rosa de las Palmas | 27 | 182-213 | 94 | 11.1 (50) | | | 7.4 (50) | |  | 14.8 (50) |
| **Olmedo County** |  |  |  |  | | |  | |  |  |
| Guaijil | 20 | 114 | 2 | 5.0 (100) | | |  | |  | 5.0 (100) |
| **Pajan County** |  |  |  |  | | |  | |  |  |
| El Porvenir | 23 | 84 | 2 | 4.3 (100) | | |  | |  | 4.3 (100) |
| La Curia | 25 | 174 | 43 | 4.0 (100) | | |  | |  | 4.0 (100) |
| Limón del Procel | 21 | 248 | 1 |  | | |  | | 4.8 (0) | 4.8 (100) |
| **Pedernales County** |  |  |  |  | | |  | |  |  |
| Cholote | 22 | 31-52 | 20 | 9.1 (33.3) | | |  | | 4.5 (100) | 13.6 (67) |
| **Pichincha County** |  |  |  |  | | |  | |  |  |
| Piedra Fina | 20 | 325 | 5 | 5.0 (100) | | |  | |  | 5.0 (100) |
| **Portoviejo County** |  |  |  |  | | |  | |  |  |
| El Bejuco | 64 | 57-304 | 264 | 17.2 (52.6) | | | 17.2 (73) | |  | 29.7 (79) |
| Las Lozas Adentro | 26 | 459 | 4 | 3.8 (100) | | |  | |  | 3.8 (100) |
| **Puerto López County** |  |  |  |  | | |  | |  |  |
| Agua Blanca | 22 | 67-89 | 4 |  | | | 9.1 (0) | |  | 9.1 (0) |
| **Rocafuerte County** |  |  |  |  | | |  | |  |  |
| Danzarín | 32 | 128-154 | 134 | 18.8 (83.3) | | |  | |  | 18.8 (83) |
| **San Vicente County** |  |  |  |  | | |  | |  |  |
| Chita | 40 | 103 | 1 | 2.5 (100) | | |  | |  | 2.5 (100) |
| Humedal | 25 | 335 | 25 |  | | |  | | 4.0 (100) | 4.0 (100) |
| Rio Canoa | 45 | 69-100 | 7 | 4.4 (100) | | |  | |  | 4.4 (100) |
| **Santa Ana County** |  |  |  |  | | |  | |  |  |
| Estero de Noche Arriba | 32 | 115-169 | 74 | 12.5 (100) | | |  | |  | 12.5 (100) |
| Sasay | 44 | 102 | 22 | 2.3 (100) | | |  | |  | 2.3 (100) |
| Tablada El Moral | 23 | 441 | 12 | 8.7 (50) | | |  | |  | 8.7 (50) |
| **Sucre County** |  |  |  |  | | |  | |  |  |
| Pechiche Abajo | 21 | 420 | 1 | 4.8 (100) | | |  | |  | 4.8 (100) |
| San Ramón | 57 | 76-210 | 16 | 8.8 (83.3) | | |  | | 1.8 (100) | 10.5 (100) |
| **TOTAL** | **2097** | **31-459** | **1380** | **3.5 (86.3)** | | | **1.0 (50)** | | **0.4 (62.5)** | **4.5 (77)** |
| Communities where no infested domiciles were found: **El Carmen County** [10 de Agosto (18), Cajones El Achiote (31), La Brava (16), Palmeras Unidas (16)]; **San Vicente County** [Ambache Cico (9), El Remojo (6), La Mocora (21)]; **Bolívar County** [Balza Abajo (28)]; **Chone County** [Balzar (25), El Mirador (8), La Isla (18), La Pinuela (21), La Sandia (6), Las Lajas (13)]; **Jama County** [Cadialito (18), Santo Tomas (20)]; **Pajan County** [Caña Brava (24)]; **Montecristi County** [Cerro Copetón (17), Las Cruces (9)]; **Pedernales County** [Cheve Arriba (56), Cheve en Medio (24), La Mina (19), Tabiazo (41), Tomas Chila (21)]; **Flavio Alfaro County** [Chila (28), Rio Javier (38)], San Roque (13); **Sucre County** [El Toro (16), La Envidia (29), Santa Rosa (29), Santa Rosa (52), Roma (21)]; **Puerto López County** [Guale (8), Rio Blanco (20), San Isidro (22), Vueltas Largas (10)]; **24 de Mayo County** [Ladera de Noboa (24)]; Jipijapa County [Palmital (12)]; **Olmedo County** [San Roque (31)]; **Rocafuerte County** [Sosote (21)];**Pichincha County** [(Valarezo (27)]. | | | | | | | | | | |
|  | | | | | | | | | | |
